# Neurosensory development and cell fate determination in the human cochlea

**DOI:** 10.1186/1749-8104-8-20

**Published:** 2013-10-16

**Authors:** Heiko Locher, Johan HM Frijns, Liesbeth van Iperen, John CMJ de Groot, Margriet A Huisman, Susana M Chuva de Sousa Lopes

**Affiliations:** 1 Department of Anatomy and Embryology, Leiden University Medical Center, T-01-032, Einthovenweg 20, 2333 ZC Leiden, the Netherlands; 2 Department of Otorhinolaryngology, Leiden University Medical Center, Leiden, the Netherlands

**Keywords:** Human, Fetus, Cochlea, SOX transcription factors, Hair cells, Spiral ganglion, Innervation, Peripherin

## Abstract

**Background:**

Hearing depends on correct functioning of the cochlear hair cells, and their innervation by spiral ganglion neurons. Most of the insight into the embryological and molecular development of this sensory system has been derived from animal studies. In contrast, little is known about the molecular expression patterns and dynamics of signaling molecules during normal fetal development of the human cochlea. In this study, we investigated the onset of hair cell differentiation and innervation in the human fetal cochlea at various stages of development.

**Results:**

At 10 weeks of gestation, we observed a prosensory domain expressing SOX2 and SOX9/SOX10 within the cochlear duct epithelium. In this domain, hair cell differentiation was consistently present from 12 weeks, coinciding with downregulation of SOX9/SOX10, to be followed several weeks later by downregulation of SOX2. Outgrowing neurites from spiral ganglion neurons were found penetrating into the cochlear duct epithelium prior to hair cell differentiation, and directly targeted the hair cells as they developed. Ubiquitous Peripherin expression by spiral ganglion neurons gradually diminished and became restricted to the type II spiral ganglion neurons by 18 weeks. At 20 weeks, when the onset of human hearing is thought to take place, the expression profiles in hair cells and spiral ganglion neurons matched the expression patterns of the adult mammalian cochleae.

**Conclusions:**

Our study provides new insights into the fetal development of the human cochlea, contributing to our understanding of deafness and to the development of new therapeutic strategies to restore hearing.

## Background

The cochlea houses two of the main cell types responsible for hearing: the hair cells and the spiral ganglion neurons (SGNs). Damage to the cochlea is usually associated with degeneration and irreversible loss of these cell types, which ultimately leads to permanent sensorineural hearing loss, the most common type of deafness [1,2]. In order to develop new therapeutic strategies, it is essential to have a better understanding of the normal molecular development of the human cochlea.

In the human embryo, the otic placode invaginates to form the otic vesicle (or otocyst) during week 6 of gestation (W6), equivalent to week 4 of fetal development [3]. In the subsequent weeks, the otic vesicle develops into both the vestibular organs and the cochlea. The cochlear duct spirals around a central axis, and reaches its final 2.5 turns by W10 to W11 [4,5]. At this stage, the epithelial lining of the cochlear duct is still undifferentiated. In mice, a dedicated area within the epithelium of the cochlear duct floor has been identified as the 'prosensory domain’ [6]; this contains the precursors to the inner hair cells (IHCs), the outer hair cells (OHCs), and various types of surrounding supporting cells, which together form the organ of Corti (OC) [7,8]. The prosensory domain is flanked by two other domains: Kölliker’s organ (KO) and the future outer sulcus. Although the prosensory domain has not been formally described in humans, hair cells are first visible by W12 in the human fetus in the region where the OC will form [5]. The OC reaches its gross adult morphology around W20, which corresponds to the onset of auditory function [9-11].

Development of the prosensory domain into the OC coincides with the establishment of highly specialized innervation patterns by afferent type I and type II SGNs. In humans, multiple type I SGNs innervate single IHCs in a 'radial’ organization, and make up 90 to 95% of the total population of SGNs, whereas single type II SGNs contact multiple OHCs in a 'spiral’ organization [12]. In mice, SGNs project to both IHCs and OHCs until 6 to 7 days after birth, when a clear distinction between type I and type II ganglion neurons takes place, just prior to the onset of hearing (post-natal week 2) [13,14]. In humans, penetration of the SGN neurites into the cochlear neuroepithelium has been observed earlier than the first differentiation of hair cells by electron microscopy [5]. The peripheral neurites of the SGNs penetrate the basal turn around W11, and in the following weeks find their way to the developing hair cells and shape their synaptic connections [5,15]. However, the separation of type I and type II SGNs has not been investigated in humans.

Here, we investigated both the dynamics of development of human cochlear hair cells and their innervation. The spatial and temporal dynamics of hair cell differentiation was determined by examining the expression of three members of the SOX family, a group of genes involved with cell fate decisions: *SOX2*, *SOX9*, and *SOX10*. An example of early cell fate specification in the cochlear duct epithelium is the spatially restricted expression of SOX2 to the cells of the prosensory domain [8]. The functional importance of the SOX2 transcription factor in normal cochlear development is further illustrated by failure of the prosensory domain establishment in loss-of-function conditions [8], and underdevelopment of hair cells in gain-of-function conditions [16]. *Sox9* and *Sox10* are known to be expressed in the otic placode and the otic vesicle in frog and chick [17-20]. In mice, SOX9 is also expressed in the otic placode and otic vesicle and controls invagination [21], and both SOX9 and SOX10 have been found in the mouse cochlear duct epithelium [22-26]. Interestingly, in mice, Sox9 and Sox10 are downregulated before or upon hair cell differentiation, whereas Sox2 is downregulated gradually, although all three *Sox* genes remain expressed in the underlying supporting cells in the OC [8,22,23].

In humans, SOX2, SOX9, and SOX10 are likely to play an important role in cochlear development, as mutations in all three genes have been shown to cause sensorineural hearing loss [27-29]. However, although SOX10 expression has been reported in the human otic vesicle [30], expression patterns of these SOX transcription factors, and their dynamics upon hair cell differentiation, have not previously been determined in the (developing) human cochlea. In addition, the innervation of the IHCs and OHCs was in the current study investigated by comparing the dynamics of expression of Peripherin (PRPH), an intermediate filament protein that is expressed in type II SGNs, both in adult mouse and adult human cochleae [13,31], along with the expression of class III β-Tubulin (TUBB3), a general SGN marker. The comprehensive description of the molecular and morphological events taking place in the cochlea as functional hearing develops may benefit the development of strategies for cochlear repair.

## Results

### The human prosensory domain is SOX2-positive

**Figure 1:**
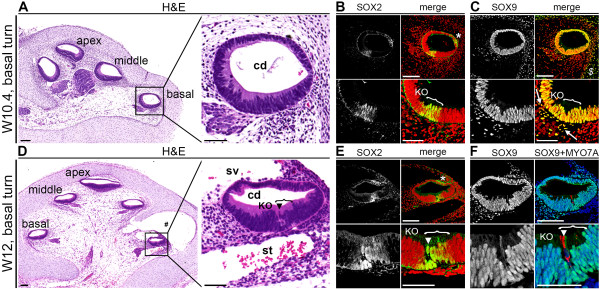
**SOX2 and SOX9 expression in human fetal cochlea around the onset of first hair cell differentiation. (A)** Hematoxylin and eosin (H&E) staining of a cochlea at W10.4 (week 10 and 4 days) with higher magnification (right panel) of the basal turn cochlear duct. **(B)** Basal turn of a W10.4 cochlea immunostained for SOX2, and magnification of the prosensory domain (bottom panels). Cell nuclei were visualized with TO-PRO-3 (red). **(C)** Basal turn of a W10.4 cochlea immunostained for SOX9, and magnification of the prosensory domain (bottom panels). Cell nuclei were visualized with TO-PRO-3 (red). **(D)** H&E staining of a W12 (week 12) cochlea with higher magnification (right panel) of the basal turn cochlear duct. **(E)** Basal turn of a W12 cochlea immunostained for SOX2, and magnification of the prosensory domain (bottom panels). Cell nuclei were visualized with TO-PRO-3 (red). **(F)** Basal turn of a W12 cochlea immunostained for SOX9 (green) and MYO7A (red), and magnification of the prosensory domain (bottom panels). Cell nuclei were visualized with DAPI (blue). #Tissue artifact; *, cytoplasmic SOX2 staining in the cochlear duct; $, SOX9 staining in the otic capsule; bracket, the prosensory domain; white arrow, Schwann cells of the spiral ganglion; arrowhead, inner hair cell. Abbreviations: cd, cochlear duct; KO, Kölliker’s organ; sv, scala vestibuli; st, scala tympani. Scale bars = 100 μm (all lower magnifications) or 50 μm (all higher magnifications).

To determine whether a prosensory domain also exists during human development, we investigated the expression of SOX2 at W10.4 (week 10 and 4 days), a stage when the cochlear duct epithelium showed no clear morphological hair cell specification (Figure 1A). At this point, nuclear SOX2 expression was already restricted to the human prosensory domain (Figure 1B) and no expression was visible in other parts of the cochlear duct, except for cytoplasmic SOX2 expression in the lateral wall of the cochlear duct epithelium (Figure 1B, asterisk). At W10.4, SOX9 not only overlapped with SOX2 in the prosensory domain, but showed uniform nuclear expression in all cells of the cochlear duct epithelium, similar to that described in the developing mouse cochlea [23]. SOX9 was also expressed in the Schwann cells of the adjacent spiral ganglion (Figure 1C) and in the cartilage cells of the otic capsule (Figure 1C).

### Differentiating cochlear hair cells downregulated SOX9 and SOX10, followed by SOX2

At W12, the openings of the scala vestibuli and the scala tympani were observed, respectively, above and beneath the basal turn of the cochlear duct (Figure 1D). The first morphological signs of hair cell differentiation were then visible exclusively in the basal turn, as a row of single cells that emerged facing the luminal aspect of the SOX2-positive prosensory domain (Figure 1D, E). Immunostaining for myosin VIIA (MYO7A), a marker of hair cells, confirmed this lineage specification (Figure 1F). Based on the position of the first hair cells at the border between the prosensory domain (SOX2-positive) and Kölliker’s organ (SOX2-negative), we identified these cells as IHCs (Figure 1E) and found that lineage specification to IHCs coincided with downregulation of SOX9 (Figure 1F).

**Figure 2:**
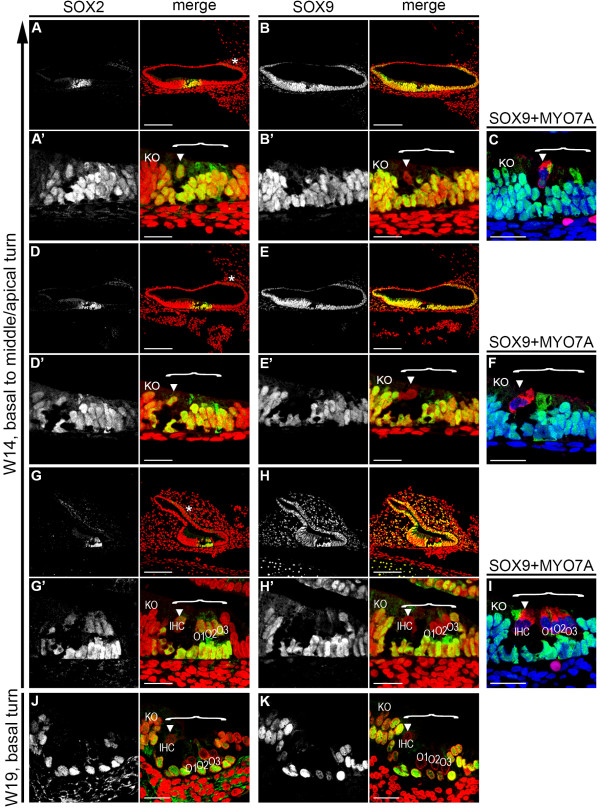
**SOX2 and SOX9 expression during development of the human organ of Corti (OC). (A,B)** Apical turn of a W14 cochlea immunostained for **(A)** SOX2 and **(B)** SOX9 and magnification of the prosensory domain/OC **(A′,B′)**. Cell nuclei were visualized (red) with TO-PRO-3. **(C)** The prosensory domain/OC in the apical turn of a W14 cochlea immunostained for SOX9 (green) and MYO7A (red). Cell nuclei were visualized with DAPI (blue). **(D,E)** Middle turn of a W14 cochlea immunostained for **(D)** SOX2 and **(E)** SOX9, and **(D′,E′)** magnification of the prosensory domain/OC. Cell nuclei were visualized with TO-PRO-3 (red). **(F)** The prosensory domain/OC in the middle turn of a W14 cochlea immunostained for SOX9 (green) and MYO7A (red). Cell nuclei were visualized with DAPI (blue). **(G,H)** Basal turn of a W14 cochlea immunostained for **(G)** SOX2 and **(H)** SOX9, and **(G′,H′)** magnification of the prosensory domain/OC. **(I)** The prosensory domain/OC in the basal turn of a W14 cochlea immunostained for SOX9 (green) and MYO7A (red). Cell nuclei were visualized with DAPI (blue). **(J,K)** Basal turn of a W19 cochlea immunostained for **(G)** SOX2 and **(H)** SOX9. Cell nuclei were visualized with TO-PRO-3 (red). *Cytoplasmic SOX2 staining in the cochlear duct; bracket, the prosensory domain/OC; arrowhead, inner hair cell. Abbreviations: KO, Kölliker’s organ; IHC, inner hair cell; O1, first row of outer hair cells; O2, second row of outer hair cells; O3, third row of outer hair cells. Scale bars = **(A–K)** 50 μm or **(A**′**–H**′**)** 20 μm.

At W14 (2 weeks later) the SOX2-positive prosensory domain is developing into the OC, with maturation progressing in a basal-to-apical gradient. In the current study we found that solitary IHCs were differentiated in the apical and middle turns, whereas in the basal turn, not only were the IHCs visible, but all three rows of OHCs (O1, O2, and O3) had formed (Figure 2A-I). In general, three rows of OHCs were present; however, occasionally four rows of outer hair cells were detected. Upon hair cell specification, as confirmed by MYO7A expression, both IHCs and OHCs showed specific downregulation of SOX9 (Figure 2A-I), whereas the supporting cells underneath both IHCs and OHCs remained positive for both SOX2 and SOX9 (Figure 2A-I).

**Figure 3:**
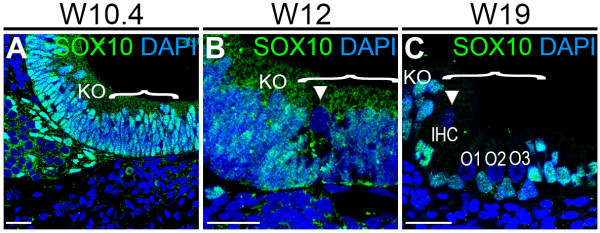
**SOX10 expression during human organ of Corti (OC) development. (A-C)** Basal turn of a cochlea immunostained for SOX10 (green) at **(A)** W10.4, **(B)** W12 and **(C)** W19. Cell nuclei were visualized with DAPI (blue). Bracket, the prosensory domain/OC; arrowhead, inner hair cell. Abbreviations: KO, Kölliker’s organ. Scale bars = 20 μm.

In the basal turn of the W19 cochlea, all IHCs and OHCs had become negative for both SOX2 and SOX9, in contrast to the supporting cells in the OC and in some of the adjacent cells in Kölliker’s organ, which expressed SOX2 (Figure 2J,K), acquiring the mature SOX2/SOX9 expression pattern seen in the mouse OC [23,32], which has yet to be investigated in the adult human cochlea. In the developing hair cells, the dynamics of SOX10 and SOX9 expression were identical (Figure 3). Downregulation of SOX10 coincided directly with the first hair cell specification at W12, and expression was maintained in supporting cells (Figure 3).

**Figure 4:**
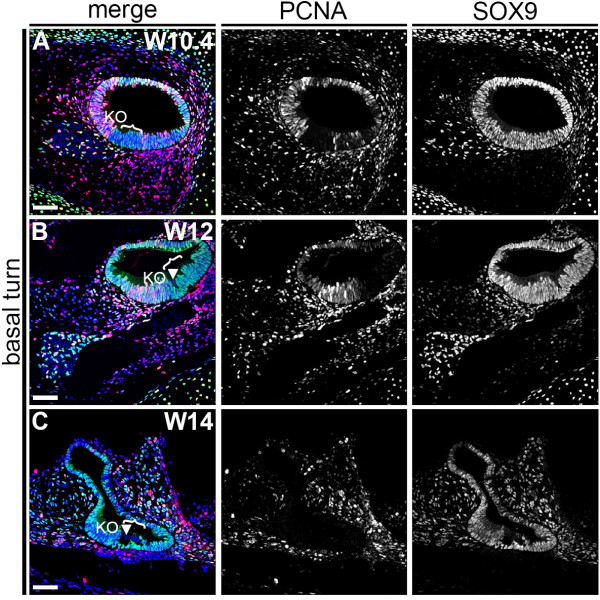
**Proliferating cell nuclear antigen (PCNA) and SOX9 expression in the basal turn of W10.4 to W14 human fetal cochlea. (A-C)** Basal turn of a cochlea immunostained for PCNA (red) and SOX9 (green) at **(A)** W10.4, **(B)** W12 and **(C)** W14. Cell nuclei were visualized with DAPI (blue). Bracket, the prosensory domain/OC; arrowhead, inner hair cell. Abbreviations: KO, Kölliker’s organ. Scale bars = 40 μm.

At W12 to W19, in the developing OC, either only the IHCs or the full set of IHCs and OHCs (O1, O2, and O3) were visible. Therefore, we next investigated whether the OHCs could be derived from the first differentiated IHCs by cell division. However, after performing immunostaining for proliferating cell nuclear antigen (PCNA), a marker of cycling cells, we found that all cells of the prosensory domain had exited the cell cycle at this stage (W10 to W12) (Figure 4A,B). This strongly suggested that cells in the prosensory domain/OC do not proliferate, supporting the idea that the OHCs differentiate from cells in the prosensory domain, but are not progeny of the IHCs. At W14, most cells in the cochlear duct, including the OC cells, were PCNA-negative (Figure 4C).

### Innervation of the cochlear duct by PRPH-positive neurites precedes hair cell differentiation

**Figure 5:**
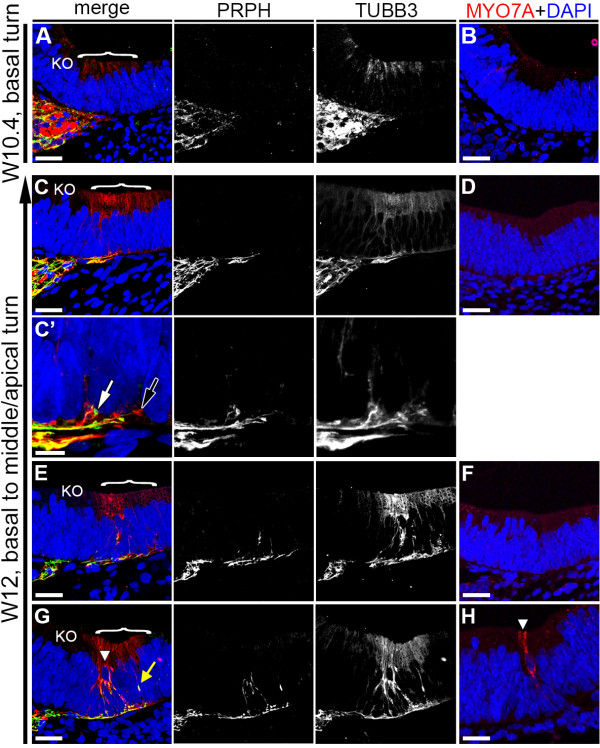
**Dynamics of Peripherin (PRPH)-positive and class III β-Tubulin (TUBB3)-positive neurites during first hair cell differentiation. (A,B)** Basal turn of a W10.4 (week 10 and 4 days) cochlea immunostained for **(A)** PRPH (green) and TUBB3 (red) and **(B)** MYO7A (red). **(C,D)** Apical turn of a W12 cochlea immunostained for **(C)** PRPH and TUBB3 and MYO7A (D). **(C′)** Magnification of the prosensory domain in **(C)**. **(E,F)** Middle turn of a W12 cochlea immunostained for **(E)** PRPH and TUBB3 and **(F)** MYO7A. **(G,H)** Basal turn of a W12 cochlea immunostained for **(G)** PRPH and TUBB3 and **(H)** MYO7A. Cell nuclei were visualized with DAPI (blue). Bracket, the prosensory domain/OC; arrowhead, inner hair cell; white arrow, PRPH-positive growth cone; black arrow, TUBB3-positive growth cone; yellow arrow, PRPH/TUBB3-positive neurite. Abbreviations: KO, Kölliker’s organ. Scale bars = **(A-C**, **D-H)** 20 μm or **(C**′**)** 5 μm.

At W10.4, both PRPH-positive and TUBB3-positive neurites were present at the distal end of the spiral ganglion in the basal turn directly beneath the prosensory domain, but these neurites did not innervate into the epithelium of the cochlear duct (Figure 5A). As there are no hair cells yet at this stage, no cells expressed MYO7A (Figure 5B).

As at W10.4, both PRPH-positive and TUBB3-positive neurites where still located directly below the prosensory domain in the W12 apical turn, (Figure 5C). However, high magnification scanning revealed the presence of PRPH-positive and TUBB3-positive growth cones extending a few micrometers into the cochlear epithelium (Figure 5C′, white and black arrows, respectively), suggesting that in humans, neurites penetrate the basement membrane prior to signs of hair cell differentiation, as confirmed by the lack of MYO7A (Figure 5D).

In the W12 middle turn, innervation by both PRPH and TUBB3 positive neurites advanced further into the epithelium (Figure 5E). These neurites penetrated the basement membrane at multiple positions directly below the reorganizing prosensory domain, and seemed directed predominantly toward one specific cell type, most probably the first future hair cell to emerge, as the prosensory domain remained MYO7A-negative (Figure 5F).

In the W12 basal turn, the neurites progressed upwards along different routes and contacted the base of the first differentiated MYO7A-positive hair cells, identified here as IHCs, at multiple positions along its basal side (Figure 5G,H). Many neurites seemed to express both PRPH and TUBB3. Strikingly, single neurites positive for both PRPH and TUBB3 invaded the epithelium at a more lateral position, at the site of the future OHCs (Figure 5G, yellow arrow), suggesting that innervation into the future OHC area precedes OHC differentiation, just as innervation into the IHC area precedes IHC differentiation.

### PRPH-positive neurites become restricted to the OHC by W20.3

**Figure 6:**
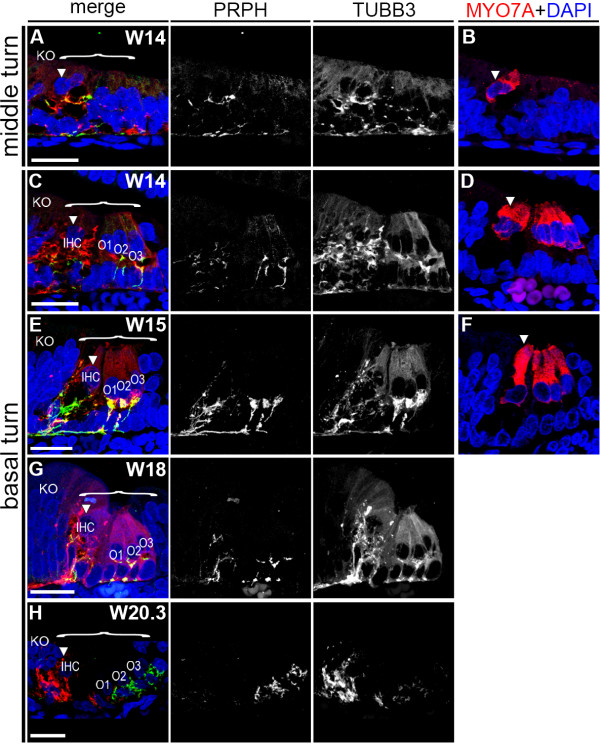
**Dynamics of Peripherin (PRPH)-positive and class III β-Tubulin (TUBB3)-positive neurites during organ of Corti (OC) maturation. (A,B)** Middle turn of a W14 (week 14) cochlea immunostained for **(A)** PRPH (green) and TUBB3 (red) and **(B)** MYO7A (red). **(C,D)** Basal turn of a W14 cochlea immunostained for **(C)** PRPH and TUBB3 and **(D)** MYO7A. **(E,F)** Basal turn of a W15 cochlea immunostained for **(E)** PRPH and TUBB3 and **(F)** MYO7A. **(G)** Basal turn of a W18 cochlea immunostained for PRPH and TUBB3. **(H)** Basal turn of a W20.3 cochlea immunostained for PRPH and TUBB3. Cell nuclei were visualized with DAPI (blue). Bracket, the prosensory domain/OC; arrowhead, inner hair cell. Abbreviations: KO, Kölliker’s organ; IHC, inner hair cell; O1, first row of outer hair cells; O2, second row of outer hair cells; O3, third row of outer hair cells. Scale bars = 20 μm.

At W14, the middle turn showed only IHCs (Figure 6A,B), whereas at W15, the middle turn showed the full complement of IHCs and OHCs. In both stages, the full complement of IHCs and OHCs was visible in the basal turn (Figure 6C-F). At W14 to W15, there were abundant PRPH-positive and TUBB3-positive neurites targeting basically all the hair cells (IHCs and OHCs) in the middle and basal turns (Figure 6A-F). At W15, all OHCs (O1, O2, and O3) were innervated by neurites that followed the basement membrane, and extended upwards in between supporting cells, and these neurites ended in a calyx-like cluster (Figure 6E). Three-dimensional (3D) reconstructions showed that at W14 and W15, in contrast to W12, some of these PRPH-positive neurites contacting the OHCs had already acquired the characteristic 'spiral’ organization, rather than penetrating the basement membrane perpendicularly toward the nearest OHC (see Additional files 1, 2 and 3).

By W18, innervation by PRPH-positive neurites to the IHCs gradually diminished (Figure 6G) and by W20.3, PRPH–positive neurites innervated the OHCs exclusively (Figure 6H), similar to the specific innervation pattern of the adult cochlea [13,31]. 3D reconstruction revealed the increased to complete 'spiral’ organization of the PRPH–positive neurites innervating to the OHC (see Additional files 4 and 5).

### Ubiquitous PRPH expression becomes restricted to type II SGNs at W18

**Figure 7:**
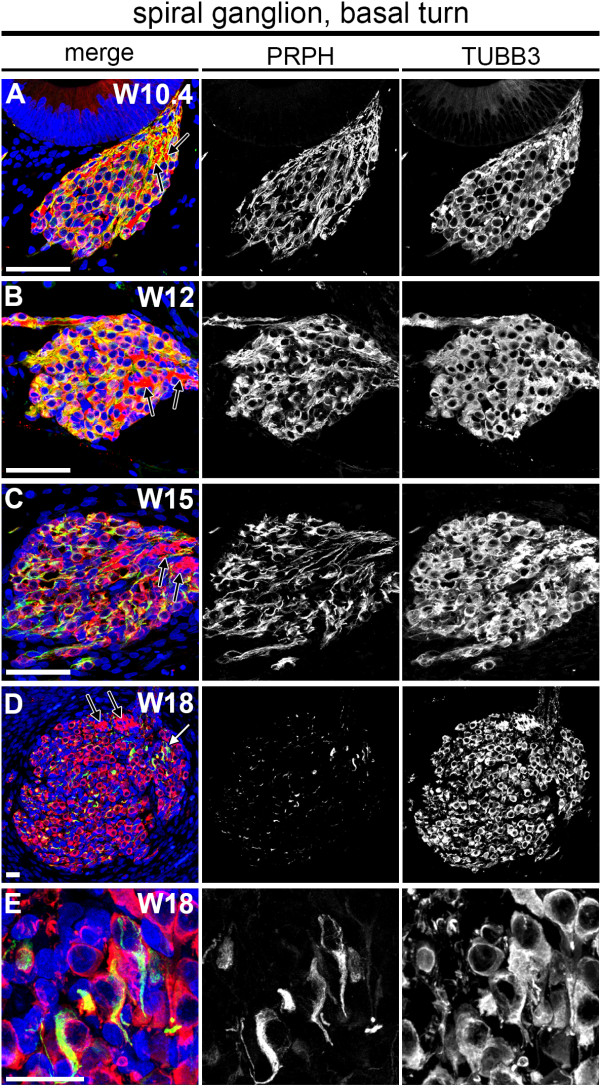
**Dynamics of Peripherin (PRPH) and class III β-Tubulin (TUBB3) expression in the human fetal spiral ganglion. (A-E)** Basal turn spiral ganglion immunostained for PRPH (green) and TUBB3 (red) at **(A)** W10.4 (week 10 and 4 days), **(B)** W12, **(C)** W15 and **(D)** W18. **(E)** Magnification of the basal turn spiral ganglion marked with a white arrow in **(D)**. Cell nuclei were visualized with DAPI (blue). White arrow, PRPH-positive spiral ganglion neurons; black arrow, PRPH-negative and TUBB3-positive nerve fiber bundles. Scale bars = 20 μm.

To further understand the separation of type I and type II SGNs in humans, we mapped the dynamics of PRPH expression in the developing spiral ganglion adjacent to the developing prosensory domain/OC. At W10 to W15, both PRPH and TUBB3 expression was visible throughout the spiral ganglion in the basal turn (Figure 7A-C), with PRPH expression reaching a maximum at W12. PRPH-negative but TUBB3-positive nerve fibers were consistently found near the distal end of the ganglion, possibly representing the efferent, intra-ganglionic spiral bundles (Figure 7, black arrows).

Strikingly, by W18, the spiral ganglion was found to be largely devoid of PRPH (Figure 7D,E). Only some SGNs strongly expressed PRPH (Figure 7D). Together with the observation that PRPH-positive neurites become confined to the OHCs and their increase in a spiral orientation, this suggests the emergence of *bona fide* type II SGNs at W18 to W20 in humans.

### Initial innervation of the cochlear duct is not conserved between mouse and human

**Figure 8:**
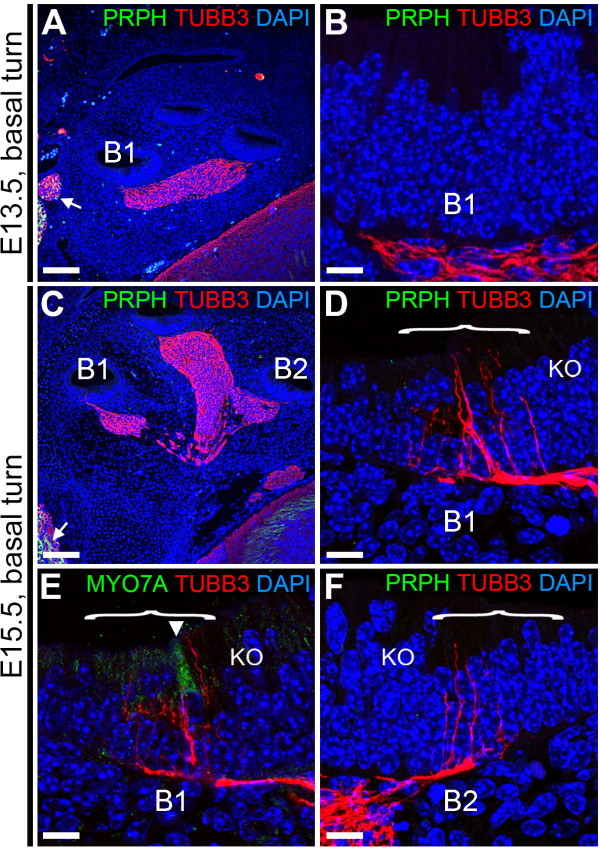
**Peripherin (PRPH) and class III β-Tubulin (TUBB3) expression in embryonic day 13.5 (E13.5) and E15.5 mouse cochlea. (A,B)** E13.5 mouse cochlea immunostained **(A)** for PRPH (green) and TUBB3 (red) and **(B)** magnification of the prosensory domain of basal turn B1. **(C-F)** E15.5 mouse cochlea **(C)** immunostained for PRPH and TUBB3, **(D)** magnification of the prosensory domain of basal turn B1, **(E)** magnification of the prosensory domain of basal turn B1 immunostained for TUBB3 (red) and MYO7A (green), and **(F)** magnification of the prosensory domain of basal turn B2 immunostained for PRPH (green) and TUBB3 (red). Cell nuclei were visualized with DAPI (blue). Bracket, the prosensory domain; arrowhead, inner hair cell. Abbreviations: KO, Kölliker’s organ. Scale bars = **(A,C)** 100 μm or **(B, D-F)** 10 μm.

We have described here that innervation of the cochlear duct in humans started around W12 and involved the simultaneous penetration by PRPH-positive and TUBB3-positive neurites into the cochlear epithelium. To complement previous mouse studies that focused specifically on the expression of PRPH at late and post-natal stages of development (embryonic day 18 (E18) to post-natal day 7 (P7)) [13,14], we investigated the expression dynamics of PRPH at the start of penetration of SGN neurites into the mouse cochlear duct, and the onset of hair cell differentiation. At E13.5, TUBB3 was present only beneath the prosensory domain of the basal turn of the cochlear duct, as we found in humans at W10 (Figure 8A,B). However, even though some of the neural structures outside the cochlea showed PRPH positivity (Figure 8A, white arrow), as well as type II SGNs in adult mouse cochlea that had been processed identically in order to confirm correct immunoreactivity (data not shown), the spiral ganglia in the cochlea of E13.5 mice were completely PRPH-negative, in contrast to our findings in the human cochlea at W10. Two days later, at E15.5, innervation by TUBB3-positive neurites was clearly detected in the basal turn, but innervation by PRPH-positive neurites was still not seen (Figure 8C-F). It should be noted that in mice (and humans), innervation of the epithelium preceded hair cell differentiation, as only a single MYO7A-positive IHC was detected in the lower (B1) basal turn (Figure 8E), but none was seen in the upper (B2) basal turn, where neurites had already innervated into the epithelium.

## Discussion

### Differentiation of the IHC at W12

Using transmission electron microscopy, Pujol and Lavigne-Rebillard previously showed that the onset of first hair cell differentiation in the human fetal cochlea starts in W12 of gestation (that is, week 10 of fetal development) [5]. In the current study, we consistently observed epithelial reorganization in the prosensory domain concurrently with SOX9 downregulation and MYO7A expression in a single row of cells in the prosensory domain of the basal turn, indicating first (inner) hair cell differentiation at W12. However, in one cochlea from W11.4, there were identical changes in marker expression in one out of three sections of the basal turn (see Additional file 6: Figure S1), even though previous ultrastructural investigations had reported an undifferentiated poly-layered epithelium [5], suggesting that hair cell differentiation might already start at the end of W11.

### Do SOX2 and SOX9/SOX10 differentially regulate hair cell differentiation?

In mammals and other vertebrates, it has been shown that hair cell differentiation is restricted to cells of the SOX2-positive prosensory domain [33]. Our data are in complete agreement with these observations, as we found that the developing human cochlea at W10.4 exhibited a SOX2-positive prosensory domain in which hair cell precursors subsequently differentiated into IHCs and OHCs in a radial and longitudinal gradient. Sox2 has been shown to act on Atoh1, the key transcription factor for hair cell differentiation [16]. Expression of Atoh1, and thereby hair cell fate commitment, is also under strict control of the Notch pathway [34]. Interestingly, there was downregulation of SOX9 and SOX10 coincident with the moment of first hair cell commitment, which was followed several weeks later by downregulation of SOX2. The same sequence of events for SOX9 and SOX2 has been previously reported in the developing mouse cochlear duct [23]. Together with our observations, this supports a distinct role for SOX9/SOX10 and SOX2 in hair cell fate commitment, and an evolutionarily conserved mechanism of hair cell differentiation between mice and humans. It is known from studies in other tissues that Sox9 is directly controlled by Notch activity, for example in the developing nervous system, where it is involved in glial versus neuronal cell fate [35], and in the developing pancreas, where Sox9 and Notch regulate endocrine versus ductal cell fate [36]. Sox9 could possibly affect hair cell versus supporting cell fate in a similar, Notch-dependent manner. Furthermore, we observed the expression of SOX2 by supporting cells in the human cochlea up to the final stage we investigated, at W20.3. As it is currently thought that Sox2 expression in supporting cells is linked to a dormant potential of hair cell differentiation [37], this validates (animal) research focusing on this pathway to restore hearing. SOX2 expression in the adult human cochlea remains to be investigated.

### Innervation dynamics of the cochlear duct by PRPH-positive neurites

Hair cell development progresses hand in hand with the arrival and shaping of afferent neurites into the cochlear duct epithelium [11]. In contrast to mice, human PRPH was expressed in SGNs prior to hair cell innervation, and the dynamics in the spiral ganglion correlated perfectly with the initial steps of innervation within the developing OC in humans. We found abundant PRPH expression at W12 and W15 in SGN cell bodies and in neurites reaching both the IHCs and OHCs. At W18, PRPH expression had become limited to cells generally located at the distal end of the spiral ganglion. It is well known that type II SGNs in the adult human cochlea are also found mainly in this area [12]. In addition, in the adult human cochlea, type II SGNs represent less than 10% of the total number of SGNs [12], and PRPH expression has been found to be restricted to this cell type [31]. In relation to our observations, it can therefore be concluded that PRPH expression becomes gradually restricted to type II SGNs by W18-W20.

### Onset of human hearing by W20?

Complete absence of PRPH-positive neurites projecting to IHCs was observed at W20. At this gestational stage, the IHCs were abundantly innervated by TUBB3-positive neurites. The spiral orientation of neurites projecting to OHCs was already found at W14 to W15, and was prominently present at W20. These observations are in line with adult expression patterns and orientation, providing further support for the timing of onset of human cochlear function, which is thought to take place around W20 [9-11].

## Conclusions

**Figure 9:**
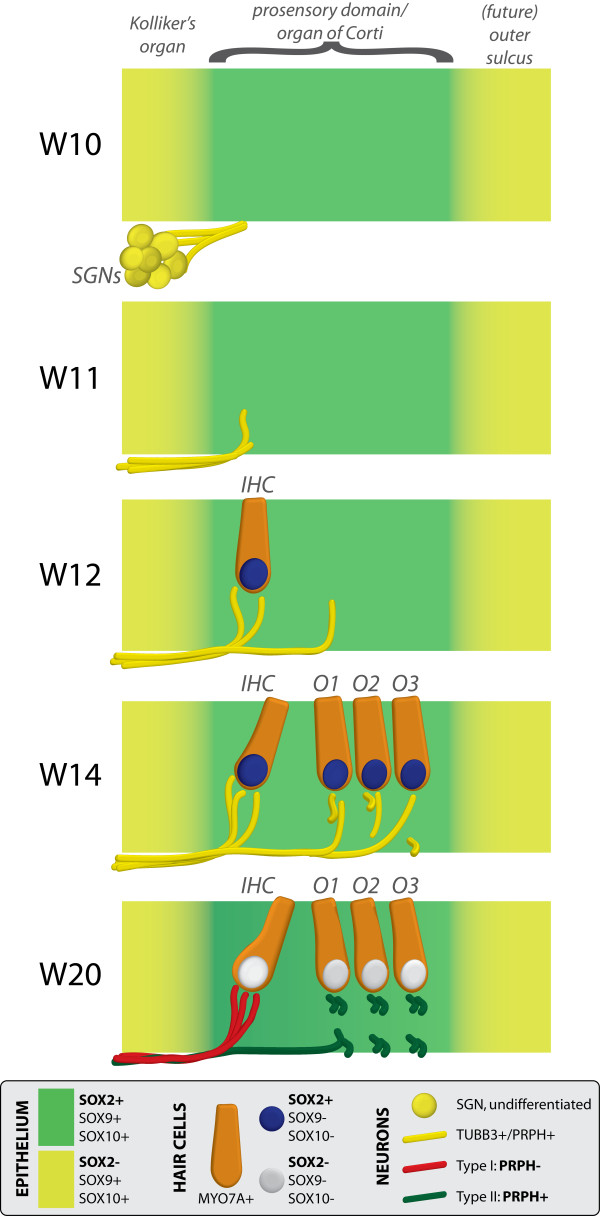
**Schematic diagram of neurosensory development in the basal turn of the human fetal cochlea.** At W10 (week 10), SOX2 identifies the prosensory domain within the SOX9/SOX10+ cochlear duct epithelium. Neurites from the adjoining TUBB3+/PRPH + SGNs do not yet penetrate into the epithelium. Penetration starts at W11, prior to hair cell differentiation. At W12, the first MYO7A+/SOX9-/SOX10-/SOX2+ (inner) hair cell can be seen, and is contacted by multiple TUBB3+ and PRPH + neurites. Penetrating neurites are also found at the location of the future OHCs. At W14, both the IHCs and OHCs have differentiated, and neurites underneath the OHCs start to run in a spiral direction. At this stage, hair cells still express SOX2. At W20, SOX2 is downregulated in all hair cells, as opposed to the other cells in the organ of Corti. PRPH expression distinguishes between type I (PRPH-) and type II (PRPH+) neurites. Abbreviations: SGN, spiral ganglion neuron; IHC, inner hair cell; O1, first row of outer hair cells; O2, second row of outer hair cells; O3, third row of outer hair cells; OHC, outer hair cell.

In conclusion, this work (summarized in Figure 9) provides some much-needed insight into the development of the human cochlea. We have shown that a SOX2-positive prosensory domain exists within the fetal human cochlear duct. Furthermore, the results presented here support the notion that SOX2 and SOX9/SOX10 may have different roles in hair cell versus supporting cell fate determination. Our investigations into hair cell innervation have shown that both TUBB3-positive and PRPH-positive neurites penetrate the basement membrane of the cochlear epithelium as early as W12 and target the subsequent developing first hair cell. This in contrast to the mouse, in which PRPH expression is detected later. Finally, we determined that already by W18 to W20, PRPH expression distinguishes between type I and type II SGNs, in contrast to mice and other rodents, in which this specialization occurs only during post-natal development of the cochlea [13,14,38]. Together, these results bring us closer to understanding the timing of some of the essential steps and the identification of some of the key molecular players during human cochlear development. Thus, we provide a basis for research focused on regeneration of the auditory system and restoration of hearing.

## Methods

### Ethics approval

The medical ethics committee of the Leiden University Medical Center approved this study (protocol 08.087), and informed consent was obtained in accordance with the WMA Declaration of Helsinki guidelines.

### Tissue samples

In total, 27 human embryonic and fetal cochleae were collected from tissue obtained by elective termination of pregnancy (by vacuum aspiration, after obstetric ultrasonography to determine gestational age in weeks and days) at various gestational stages (W10 to W20: W10, n = 4; W11, n = 1; W12, n = 4; W14, n = 4; W15, n = 2; W16, n = 1; W17, n = 4; W18, n = 4; W19, n = 2; W20, n = 1).

Time between termination and collection was kept to a minimum, ranging from one to several hours. All cochlear specimens were fixed in 4% paraformaldehyde in PBS overnight at 4°C. Cochleae obtained before W14 were dehydrated in ethanol and embedded in paraffin wax using standard procedures. Cochleae from W14 and later were decalcified for 1 to 3 weeks in 10% EDTA disodium salt (pH 7.4) (Sigma-Aldrich, St Louis, MO, USA) in distilled water at 4°C, prior to ethanol dehydration and paraffin wax embedding. Sagittal sections from E13.5 and E15.5 mouse embryos (CBA/Bl6) were a generous gift from the McLaren Laboratory (Wellcome Trust/Cancer Research UK Gurdon Institute, University of Cambridge, Cambridge, UK). For these, E0.5 was designated as the first morning with a vaginal plug, and tissue was fixed in 4% paraformaldehyde in PBS overnight at 4°C before paraffin wax embedding.

### Histology and immunofluorescence

The cochleae were sectioned (5 μm) in the sagittal plane using a RM2255 microtome (Leica Microsystems GmbH, Wetzlar, Germany). Sections were dewaxed in xylene, rehydrated in a descending ethanol series (100%, 90%, 80%, 70%), and rinsed in distilled water. Hematoxylin and eosin staining was performed by standard procedures to determine the morphology of each cochlea. For immunofluorescence, antigen retrieval was performed in 0.01 mol/l sodium citrate buffer (pH 6.0) for 12 minutes at 97°C using a microwave oven, and sections were allowed to cool to room temperature. The sections were subsequently blocked with 1% bovine serum albumin (BSA; Sigma-Aldrich) in PBS containing 0.05% Tween-20 (Promega, Leiden, the Netherlands) for 30 minutes, and incubated with primary antibodies diluted in blocking solution overnight at room temperature in a humidified chamber. The following day, the sections were incubated with secondary antibodies diluted in blocking solution for 2 hours at room temperature. Nuclei were stained with 4′,6-diamidino-2-phenylindole (DAPI; Vector Laboratories Ltd., Peterborough, UK) or TO-PRO-3 (Life Technologies, Carlsbad, CA, USA), and sections were mounted in ProLong Gold (Life Technologies). The primary antibodies used in this study were mouse anti-MYO7A (1:40; 138-1 supernatant; DSHB, Iowa City, IA, USA), rabbit anti-SOX2 (1:200; ab5603), rabbit anti-PRPH (1:200; ab1530) (both Chemicon, Temecula, CA, USA); rabbit anti-SOX9 (1:200; ab5535, Millipore Corp., Bedford, MA, USA), goat anti-SOX10 (1:50; sc-17342), mouse anti-PCNA (1:500;, ab-56) (both Santa Cruz Biotechnologies, Santa Cruz, CA, USA), and mouse anti-TUBB3 (1:200; ab78078, Abcam, Cambridge, UK). The Alexa Fluor conjugated secondary antibodies used were 488 donkey anti-mouse (1:500; A-21202), 488 donkey anti-rabbit (1:500; A-21206), 488 donkey anti-goat (1:500; A-11055), 568 donkey anti-mouse (1:500; A-10037) and 568 donkey anti-rabbit (1:500; A-10042 (all Life Technologies)). For antibody specificity controls, primary antibodies were omitted.

### Image acquisition and processing

Sections stained with hematoxylin and eosin were digitized using a Pannoramic MIDI scanner (3DHISTECH, Kisvárda, Hungary) and adjusted using Pannoramic Viewer (3DHISTECH). Confocal images were taken under a Leica TCS SP5 confocal inverted microscope (Leica Microsystems), operating with the Leica Application Suite Advanced Fluorescence software (LAS AF; Leica Microsystems). Sections were scanned throughout their full depth with Z-steps of 0.5 μm (or with a sampling density according to the Nyquist rate in the case of high magnification) and Z-projections were generated. Brightness and contrast adjustments, consistent with the image manipulation policy, were performed either in LAS AF or Adobe Photoshop CS6 (Adobe Systems Inc., San José, CA, USA). Amira (version 4.1; Visage Imaging, San Diego, CA, USA) was used for 3D reconstruction of entire Z-stacks.

## Abbreviations

3D: Three-dimensional; CO: Organ of Corti; DAPI: 4′,6-diamidino-2-phenylindole; IHC: Inner hair cell; KO: Kölliker’s organ; MYO7A: Myosin VIIA; O1: First row of outer hair cells; O2: Second row of outer hair cells; O3: Third row of outer hair cells; OHC: Outer hair cell; PCNA: Proliferating cell nuclear antigen; PRPH: Peripherin; SGN: Spiral ganglion neuron; TUBB3: Class III β-Tubulin.

## Competing interests

The authors declare that they have no competing interests.

## Authors’ contributions

HL and SMC designed the experiments. HL and LI collected and processed the specimens. HL carried out the immunohistochemistry and fluorescent microscopy. All authors analyzed and interpreted the data. HL and SMC drafted the manuscript; LI, JCMJG, MAH and JHMF revized the manuscript. All authors read and approved the final manuscript.

## Supplementary Material

Additional file 1: Movie 1 Three-dimensional reconstruction showing the PRPH-positive neurites (green) and the nucleus of the inner hair cell (blue) of the prosensory domain/developing organ of Corti in the lower basal turn at W12, (week 12) corresponding to Figure 5G. Click here for file

Additional file 2: Movie 2 Three-dimensional reconstruction showing the Peripherin (PRPH)-positive neurites (green) and the nuclei of the hair cells (blue) of the developing organ of Corti in the lower basal turn at W14 (week 14), corresponding to Figure 6C. Click here for file

Additional file 3: Movie 3 Three-dimensional reconstruction showing the Peripherin (PRPH)-positive neurites (green) and the nucleus of the inner hair cell (blue) of the developing organ of Corti in the lower basal turn at W15 (week 15), corresponding to Figure 6E. Click here for file

Additional file 4: Movie 4 Three-dimensional reconstruction showing the Peripherin (PRPH)-positive neurites (green) and the nucleus of the inner hair cell (blue) of the developing organ of Corti in the lower basal turn at W18 (week 18), corresponding to Figure 6G. Click here for file

Additional file 5: Movie 5 Three-dimensional reconstruction showing the Peripherin (PRPH)-positive neurites (green) and the nucleus of the inner hair cell (blue) of the developing organ of Corti in the lower basal turn at W20.3 (week 20.3), corresponding to Figure 6H. Click here for file

Additional file 6: Figure S1 The onset of hair cell differentiation. Confocal image of the prosensory domain within the lower basal turn of a W11.4 human fetal cochlea immunostained for MYO7A (red) and SOX9 (green). Nuclei were visualized (blue) with DAPI. Bracket, prosensory domain; arrowhead, inner hair cell. Scale bar = 20 μm. Click here for file
