# Supplementary figures and images for: Neurosensory development and cell fate determination in the human cochlea
Source: Neural Dev. 2013 Oct 16;8:20. doi: 10.1186/1749-8104-8-20 (PMC3854452; doi:10.1186/1749-8104-8-20)

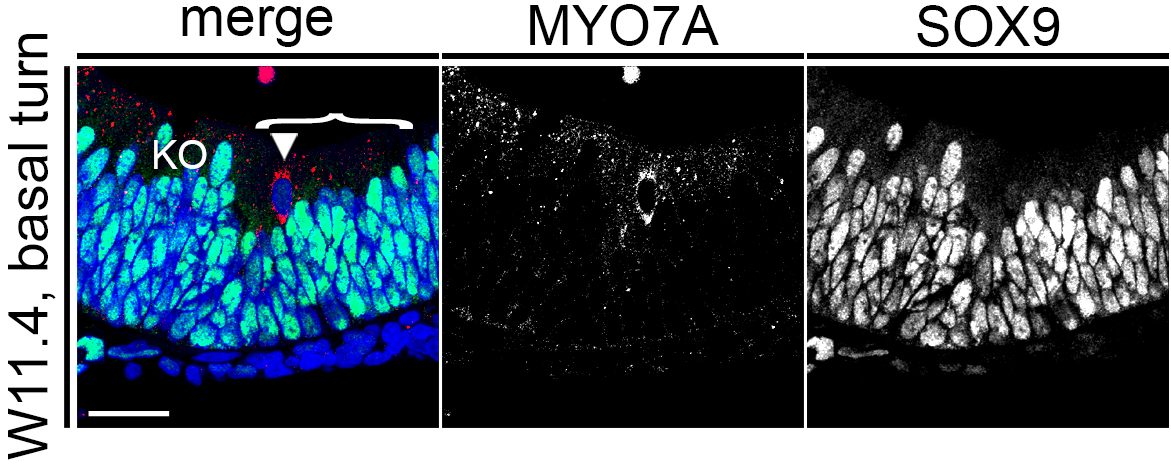

Supplement: Additional file 6: Figure S1 — The onset of hair cell differentiation. Confocal image of the prosensory domain within the lower basal turn of a W11.4 human fetal cochlea immunostained for MYO7A (red) and SOX9 (green). Nuclei were visualized (blue) with DAPI. Bracket, prosensory domain; arrowhead, inner hair cell. Scale bar = 20 μm. [file 1749-8104-8-20-S6.png]
